# Subcutaneous administration of β-hydroxybutyrate improves learning and memory of sepsis surviving mice

**DOI:** 10.1007/s13311-019-00806-4

**Published:** 2019-12-18

**Authors:** Xueqin Wang, Yaying Song, Jie Chen, Shuibing Zhang, Yuan Le, Zhongcong Xie, Wen Ouyang, Jianbin Tong

**Affiliations:** 1 Center for Experimental Medicine, Third Xiangya Hospital, Central South University, 138th Tongzipo Road, Yuelu District, Changsha, 410013 People’s Republic of China; 2 Department of Neurology, Ruijin Hospital, Shanghai Jiao Tong University School of Medicine, Shanghai, 200025 People’s Republic of China; 3 Department of Anesthesiology, Third Xiangya Hospital, Central South University, 138th Tongzipo Road, Yuelu District, Changsha, 410013 People’s Republic of China; 4 Geriatric Anesthesia Research Unit, Department of Anesthesia, Critical Care and Pain Medicine, Massachusetts General Hospital and Harvard Medical School, Charlestown, MA 02129-2060 USA; 5 Hunan Province Key Laboratory of Brain Homeostasis, Third Xiangya Hospital, Central South University, Changsha, Hunan People’s Republic of China

**Keywords:** β-hydroxybutyrate, Sepsis associated encephalopathy, Sepsis, Inflammation, MCT2, HCA2

## Abstract

Post-sepsis cognitive impairment is one of the major sequelae in sepsis survivors. Its prevention remains clinically challenging. Here we tested the effects and underlying mechanisms of exogenous β-hydroxybutyrate (BHB) on post-sepsis cognitive impairment. We found that subcutaneous administration of BHB increased survival and body weight recovery of sepsis mice and improved learning and memory of sepsis surviving mice in a cecal ligation and perforation-induced sepsis model. Additionally, the improvement of learning and memory of sepsis surviving mice was still detected even if BHB was administrated at the late stage of sepsis. In contrast, glucose solution did not show similar effects. Mechanistically, subcutaneous administration of BHB increased the BHB level of hippocampus, and limited neuroinflammation and neuroplasticity damage in sepsis mice. Intracerebroventricular administration of BHB also alleviated neuroinflammation and cognitive impairment of sepsis surviving mice. In the coculture of neurons, astrocytes, and BV2 cells (a microglial cell line), knocking down the expression of microglial HCA2 (BHB receptor) via a specific shRNA reduced the protection of BHB to lipopolysaccharide-induced inflammatory response and neuron damage more significantly than knocking down neuronal MCT2 (BHB transporter). These data showed that (1) BHB was a potential pharmacological adjunct treatment for prevention of post-sepsis cognitive impairment and (2) inhibiting neuroinflammation via HCA2 was an important mechanism.

**Electronic supplementary material:**

The online version of this article (10.1007/s13311-019-00806-4) contains supplementary material, which is available to authorized users.

## Introduction

Post-sepsis cognitive impairment is one of the major sequelae in sepsis survivors and affects 12.5-21% [1]. To date, aside from the combined use of early and appropriate antimicrobial therapy, restoration of adequate tissue/organ perfusion and timely source control at the early stage of sepsis, no specific method has been available to prevent post-sepsis cognitive impairment [2, 3]. Occurrence of post-sepsis sequelae is significantly associated with decreased life quality and decreased life independence [3, 4]. Thus, high prevalence of post-sepsis cognitive impairment remains an important problem in sepsis survivors, calling for new, simple and effective prevention methods.

Ketone body β-hydroxybutyrate (BHB) is produced in the liver and serves as an alternative energy source for the brain, heart, and skeletal muscles in mammals during states of energy deficit. In addition to its classical role of being an alternative energy source, recent studies have shown that BHB could regulate innate immune response via suppressing activation of NLRP3 inflammasome [5]. Administration of exogenous BHB could reduce reactive oxygen species production via inhibiting HDACs [6] and efficiently protecting neurons in hypoglycemic animals [7]. Interestingly, neuroinflammation and oxidative stress both played important roles in the pathogenesis of post-sepsis cognitive impairment [1, 8–13]. Additionally, the level of blood BHB decreases during sepsis [14, 15]. Thus, we think that exogenous BHB may be useful in the prevention of post-sepsis cognitive impairment.

In this study, we detected the effects and underlying mechanisms of exogenous BHB on post-sepsis cognitive impairment. We found that in contrast to a glucose solution, BHB administration increased the survival and the body weight recovery of sepsis mice and improved the learning and memory of sepsis surviving mice in a cecal ligation and perforation (CLP)-induced sepsis model. BHB administration also significantly limited neuroinflammation and neuroplasticity damage of sepsis mice. In addition, BHB administration limited lipopolysaccharides-induced neuron damage and inflammatory response via HCA2 and MCT2 in vitro. These data showed that BHB administration could prevent post-sepsis cognitive impairment.

## Materials and methods

### Animals

Experiments were performed in accordance with the guidelines for experimental animal use of the Central South University. The protocol (LLSC (LA).2015-004) was approved by the ethics committee of the Third Xiangya Hospital of Central South University. C57BL/6 male mice (8–9 weeks, 20–25g) were purchased from the Central South University (P.R. China). All of the mice were kept in an animal care facility within the Department of Laboratory Animals in Central South University under stabilized temperature (22±1°C) and lighting conditions (12-h light/12-h dark cycle). Food and water were provided with constant care and clean conditions.

In Experiment 1, the goal was to explore the effects of subcutaneous administration of BHB on SAE in a CLP-induced sepsis model. Mice were randomly divided into 5 groups: (1) sham+NS group: mice received an abdominal incision and a subcutaneous injection of pre-warmed saline (1 ml) every 6 h during the first 7 days; (2) CLP+NS group: mice received CLP surgery and a subcutaneous injection of pre-warmed saline (1 ml) every 6 h during the first 7 days; (3) CLP +BHB group: mice received CLP surgery and a subcutaneous injection of BHB (250 mg/kg [7], 1 ml) every 6 h during the first 7 days; (4) CLP +BHB(L) group: mice received CLP surgery and a subcutaneous injection of pre-warmed saline (1 ml) every 6 h during the first 3 days and BHB (250 mg/kg [7], 1 ml) every 6 h from the fouth day to the seventh day; (5) CLP +Glu group: mice received CLP surgery and a subcutaneous injection of 5% glucose solution (1 ml) every 6 h during the first 7 days.

In Experiment 2, the goal was to explore the effects of intracerebroventricular administration of BHB on neuroinflammation and cognitive function in a CLP-induced sepsis model. Mice with intracerebroventricular cannulas were randomly divided into four groups: sham+NS group, CLP+NS group, CLP +BHB group, CLP + Glu group. The treatments for each group were the same as the descriptions for the corresponding groups in Experiment 1 except for BHB administration. Here, BHB was administrated intracerebroventricularly twice a day for 7 days (250 mg/kg [7]).

### Cecal ligation and perforation (CLP) surgery model

The classical murine model of CLP was generated according to previously reported procedures [11, 16]. Briefly, mice were anesthetized with 2% sevoflurane mixed with 80–85% oxygen and placed on an electric blanket. The fur was removed and thoroughly cleaned with complex iodine. An incision of 1.5 cm was made at the midline of the abdomen. The cecum was exposed, ligated below the ileo-cecal valve, and then punctured once with a sterile 20-gauge needle. A small amount of fecal material was squeezed out of the puncture site gently before the cecum was returned back to the peritoneal cavity. Subsequently, the abdominal cavity was closed using aseptic 5–0 surgical sutures. After the surgery, all of the mice were immediately placed in a warm environment (25±1°C) for 2 days and were administered a subcutaneous injection of pre-warmed saline (1 ml) every 6 h for fluid resuscitation for 4 days. Softened food was placed onto the bedding for the mice to easily access. Buprenorphine (0.1 mg/kg) and bupivacaine (3 mg/kg) were injected subcutaneously once for postoperative pain control [11, 16]. Sham-operated mice underwent a laparotomy but without CLP. The body weight, rectal temperature, and survival of mice were carefully monitored before the endpoints or behavioral tests.

### Intracerebroventricular cannulation and BHB administration

The mice anesthetized with 2% sevoflurane were implanted stereotaxically with guide cannulas (26 gauge, 5mm, RWD life, China) into the right lateral ventricle (Bregma: -0.22 mm, dorsal-ventral: -2.35 mm, midline-right: 1 mm ). After 3 days recovery, mice were randomly divided into four groups. Intracerebroventricular injection of BHB (250 mg/kg, 5 ul) or 5% glucose solution (5%, 5 ul) were administrated by guide cannulas twice a day for 7 days.

### Novel object recognition

A novel object recognition test (NOR) was conducted during the 14^th^ and 15^th^ days after CLP in a 30×30×20-cm field following the reported protocol [17, 18]. The test consisted of two phases: a familiarization and a test phase. During the familiarization phase, two identical objects were placed equidistant from the center of the arena and equidistant from the arena walls. Each mouse was released at the edge of the arena, equidistant from two identical objects. After a free exploration of 10 min, the mouse was placed back into the home cage. Twenty-four hours after the familiarization phase, one of the objects was exchanged with a new one. The animal was placed back into the arena with the novel object and the other familiar objects for 10 min. The testing arena and objects were cleaned with 75% ethanol. The videos were scored by trained observers, and the measures of direct contact with an object (including any contact using the mouth, nose, or paw) were manually scored. Novel object preference was assessed using the time spent investigating the original object and the time spent investigating the novel object in relation to the total time spent investigating both objects.

### Barnes maze test

The mice were tested as previously described 16-19 days after CLP [11]. Briefly, the mice were trained to locate the escape hole on a Barnes maze four times per day for four days (3 min per trial and 15 min between each trial). The number of incorrect holes investigated (termed errors) and the latency to the target hole during each trial were recorded. The platform surface was cleaned with 75% ethanol before each trial to remove the odor cues. The test was performed by a technician who was blinded to the experimental design.

### Determination of beta-hydroxybutyrate levels

Under anesthesia, the blood of each mouse was collected from the right auricle into EDTA-coated tubes, and 10-20 min after blending, the blood was centrifuged for 20 min (5652 g). The supernatants were collected carefully. Additionally, the BHB concentration of plasma was measured using ELISA (Nanjing Jiancheng Bioengineering institute, E030-1) according to the manufacturer’s protocol.

Under anesthesia, the hippocampus of each mouse was removed and weighed. After adding PBS (pH 7.4), the hippocampus was homogenized and centrifuged 20 min (5652g). Finally, the supernatant was carefully collected. The BHB concentration of the supernatant was also measured using ELISA (Nanjing Jiancheng Bioengineering institude, E030-1).

### Tissue preparation

The mice were anesthetized with inhaled sevoflurane anesthesia, perfused transcardially with 0.01 M phosphate-buffered saline (PBS) and then the brains were removed. One hemisphere of each brain was used for immunostaining, and the other was used for Western blotting. The hemisphere used for immunostaining was fixed in 4% paraformaldehyde overnight at 4°C. After dehydrating with sucrose, the brains were embedded in OCT, and the transverse sections of the brain (20 μm) were serially cut using a cryostat.

From the hemisphere used for Western blotting, the hippocampus was removed and homogenized in cold lysis buffer containing protease inhibitors. After centrifuging, the supernatant of hippocampal homogenates were collected and stored at -80°C.

### Immunostaining

After washing with 0.01 M phosphate buffered saline (PBS) for 10 min, sections were incubated in 3% H_2_O_2_ for 10 min and washed in PBS three times. Then, the sections were incubated in a blocking solution (5% BSA and 0.3% Triton X-100 in 0.01 M PBS) for 1 h at room temperature. Next, the sections were incubated in primary antibodies (rabbit anti-Iba-1, lot 019–19741, 1:1000, Wako Chemical; rabbit anti-doublecortin, lot 4604 1:800, Cell Signaling Technology) overnight at room temperature. On the second day, these sections were washed with 0.01 M PBS three times and then incubated in a biotinylated secondary antibody (1:200; Vector Laboratories, USA) for 1 h at room temperature. After three washes in PBS, the sections were incubated with avidin-biotin complex reagents (ABC Elite Kit, Vector Laboratories, USA) for 1 h. Finally, the immunoreaction products were visualized using DAB kits (Beijing Zhongshan Jinqiao Biological Technology Co., Ltd, China). All of the sections were mounted, stained with hematoxylin, dehydrated, cleared, and coverslipped in a PermountTM mounting medium (Sinopharm Group Co. Ltd.). As negative controls, an adjacent series of sections were processed using the same procedures without the primary antibodies.

Based on the Iba1 staining, the percentage of activated microglia in the CA1 and DG was also determined using our reported method [11]. According to the report, an activated microglia was defined as when the cell body was bigger, pleomorphic bi- or tri-polar, or spindle/rod-shaped and when the branches were shortened, twisted, or displayed no ramification [11]. Based on the doublecortin staining, the doublecortin positive cells in the subgranular zone (SGZ) of the entire dentate gyrus (DG) were counted using a light microscope (×400). The amount of doublecortin positive cells in the five sections of the hippocampus per mouse was expressed as the cell number of the mouse [19]. Four rats were counted for each group. The data were analyzed by a trained technician who was blinded to the experimental conditions.

### Western blotting

Western blotting was used to detect the expressions of PSD-95, synaptophysin, and β-actin in the hippocampus. Briefly, frozen hippocampus tissue were homogenized in a lysis buffer containing a protease inhibitor cocktail (Roche, Germany, catalog number: P8340) and phenylmethanesulfonyl fluoride (PMSF, Sigma, USA, catalog number: p7626). The quantity of protein in the samples was determined using a BCA protein assay kit (CWbio, China) according to the manufacturer’s instructions. Equal amounts of protein samples were separated by a sodium dodecyl sulfate polyacrylamide gel electrophoresis (SDS-PAGE) and transferred to polyvinylidene fluoride membranes. After washing, the membranes were blocked with 10% skim milk in TBST buffer for 1 h and then incubated with primary antibodies (rabbit anti-PSD-95, lot ab18258, abcam; rabbit anti- synaptophysin, lot 17785-1-AP, proteintech; mouse anti-β-actin, lot 60008-1-lg, proteintech) overnight at 4°C. After three washes, the membranes were incubated with a secondary antibody (1:2000) at room temperature for 2 h. Finally, the proteins were visualized using an enhanced chemiluminescence detection kit (CWbio, China), and the intensity of each band was quantified by densitometry. The relative expression levels of protein were normalized and are presented as the ratio of the target protein (PSD-95, synaptophysin) to β-actin.

### Peripheral blood white blood cell counting

Under anesthesia, the blood of each mouse was collected from the right auricle into EDTA-coated tubes. After a three-fold dilution with saline, the blood was rapidly tested by a Mindray BC-5300 blood analyzer.

### qRT-PCR analysis

The total RNA was extracted from homogenized hippocampal tissues using Trizol Reagent (Invitrogen, USA, catalog number: CA02008). The quality of RNA was evaluated by comparing the optical densities at 260 nm and 280 nm. The RNA concentration was determined by NanoDrop (ND-1000). According to the manufacturer’s instructions, the extracted RNA was transcribed into cDNA using a Prime Script reverse transcription-PCR kit (GeneCopoeia, China, catalog number: QP001) for quantitative PCR. The primers (Table 1) for all of the assayed genes were determined using reported sequences and are listed in Table 1. The mouse GAPDH gene was used as an internal control. The data were analyzed and quantified using the 2^− ΔΔ^Ct method.

**Table 1:** Primers used for quantitative real-time PCR

Target gene	Primers	Sequence (5’-3’)
MCT2	Forward reverse	AATCTGGAGGCTGCTCTACC ATGTTTCTCTTGGCTGTTGTCAG
HCA2	Forward reverse	AGCATGAGCAAGGAATGGTG ACGTTTCCTCTGACCTCCCT
IL-1	Forward reverse	CGCAGCAGCACATCAACAAGAGC TGTCCTCATCCTGGAAGGTCCACG
TNF-α	Forward reverse	ACCCTCACACTCAGATCATCTT GGTTGTCTTTGAGATCCATGC
Iba1	Forward reverse	CCGAGGAGACGTTCAGCTAC CATCCACCTCCAATCAGGGC
GAPDH	Forward reverse	GAGGGATGCTGCCCTTACC AAATCCGTTCACACCGACCT

### Primary hippocampal neuron culture

Primary hippocampal neurons were prepared using a modified culture protocol [20]. Briefly, hippocampal from mice at E16 to E18 were dissected, the dissociated cells were set in trypsin solution (10 min at 37°C), and spread on 6-well plates coated with poly-D-lysine (Sigma, San Louis, MO). Cells were cultured in DMEM with 10% FBS at a density of 7 x 10^5^ cells/well. After 4 h of seeding, the medium was changed to a Neurobasal medium (Gibco, Carlsbad, NM) supplemented with B-27 (Gibco, Carlsbad, NM) after washing the cells once with PBS. The cells were cultured at 37°C in a humidified incubator at 37°C with 5% CO2. The hippocampal cells were used for experiments 7-10 days after seeding.

### Viral infection

The BV2 microglial cells were maintained in DMEM media containing 5% FBS and transfected with a lentivirus (LV) containing a shRNA plasmid targeting HCA2 (Genesci, Shanghai; target sequence: CAACAAATACCAGATGGTT, CCAACATTTCGTAGCCTTA, or TCAGATGAACGACGTTATT). The cells were infected in 96-well plates at multiplicities of infection (MOIs) of 200 in the presence of polybrene (8 μg/ml) until 95% transfection rate (eGFP ^+^ cells/all cells) of cells was reached. Twenty-four hours after infection, the cells were split into 24-well plates, and 72 h after infection virus-containing media were removed and replaced with normal maintenance media.

Primary neuron cells were transfected with a lentivirus (LV) containing a shRNA plasmid targeting MCT2 (Genesci, Shanghai; target sequence: TTCATTGGAGGTTTAGGATTA, CTGAAGAAAGCCAGTAAGGTA, or CAGGACGAAGTTACTGTCAAA) after a 4-day culture. The incubation time for transfection was 24 h with a 95% transfection rate(eGFP ^+^ cells/all cells) at MOIs of 10. The cells were further cultured until days 7-9 for coculture.

### Coculture of neurons, astrocytes, and BV2

Neurons, astrocytes, and BV2 cells (a microglial cell line) were cocultured as previously described with modifications [21]. After isolating the primary neurons and astrocytes, we cultured them for an appropriate amount of time. Neuron cells were cultured for 7-9 days on a round coverslip and put into six-well plates seeded with 9-12 days cultured astrocytes (90% confluence). BV2 cells were seeded on the Transwell membrane inserts with 0.4-μm pores (90% confluence) (Corning, St. Louis, MO). Thus, the three kinds of cells could be treated or collected respectively. The coculture medium contains 30% of glial cell medium(DMEM media+5% FBS) and 70% of neuronal medium(Neurobasal medium+B-27).

Twenty-four hours after co-cultured, neurons cocultured with astrocytes and BV2 cells were treated with BHB (0, 0.5, 1.0, and 1.5 mM) 6 h before and 0 h, 6 h, 12 h, and 18 h after the lipopolysaccharides (LPS, 1μg/ml) treatment. The BV2 cells and neurons were collected 24 h after the LPS treatment respectively.

### Cell survival assay (LDH)

The death of primary neurons was measured by LDH release in the culture medium. Levels of LDH release in the supernatants of cultured cells were measured using the LDH assay kit (Beyotime, Jiangsu, China). The percent cell death was calculated using the formula: % cytotoxicity = LDH release (OD492)/maximum (OD492).

### Cell viability assay (CCK-8)

The cck-8 assay kit (Dojindo, Kumamoto, Japan) was prepared according to the manufacturer’s instructions. The cck-8 assay buffer should be prepared before use, which contained 10% cck-8 test kit and 90% Neurobasal medium. Briefly, the slides with neuron cells were transferred to a new 24-well plate 24 h after LPS stimulation, and 200 μl configured cck-8 test solution was added to each well. After incubated for 2 h at 37°C, the absorbance was measured at 450 nm using a spectrometer.

### Statistical analysis

Statistical analyses were performed using Prism 5 (Graph Pad Software Inc., La Jolla, CA, USA). The data are shown as the mean ± SEM. A two-way repeated measures ANOVA was used to analyze the data from the Barnes maze test. One-way ANOVA was used to analyze the data from the open field test, Western blot, and immunostaining. The Bonferroni multiple comparison test was performed to compare selected groups when the ANOVA showed significance. The significance was set at p < 0.05.

## Results

### β-hydroxybutyrate (BHB) administration promotes survival and body weight recovery of CLP mice and improves cognitive dysfunction of CLP surviving mice

**Fig. 1:**
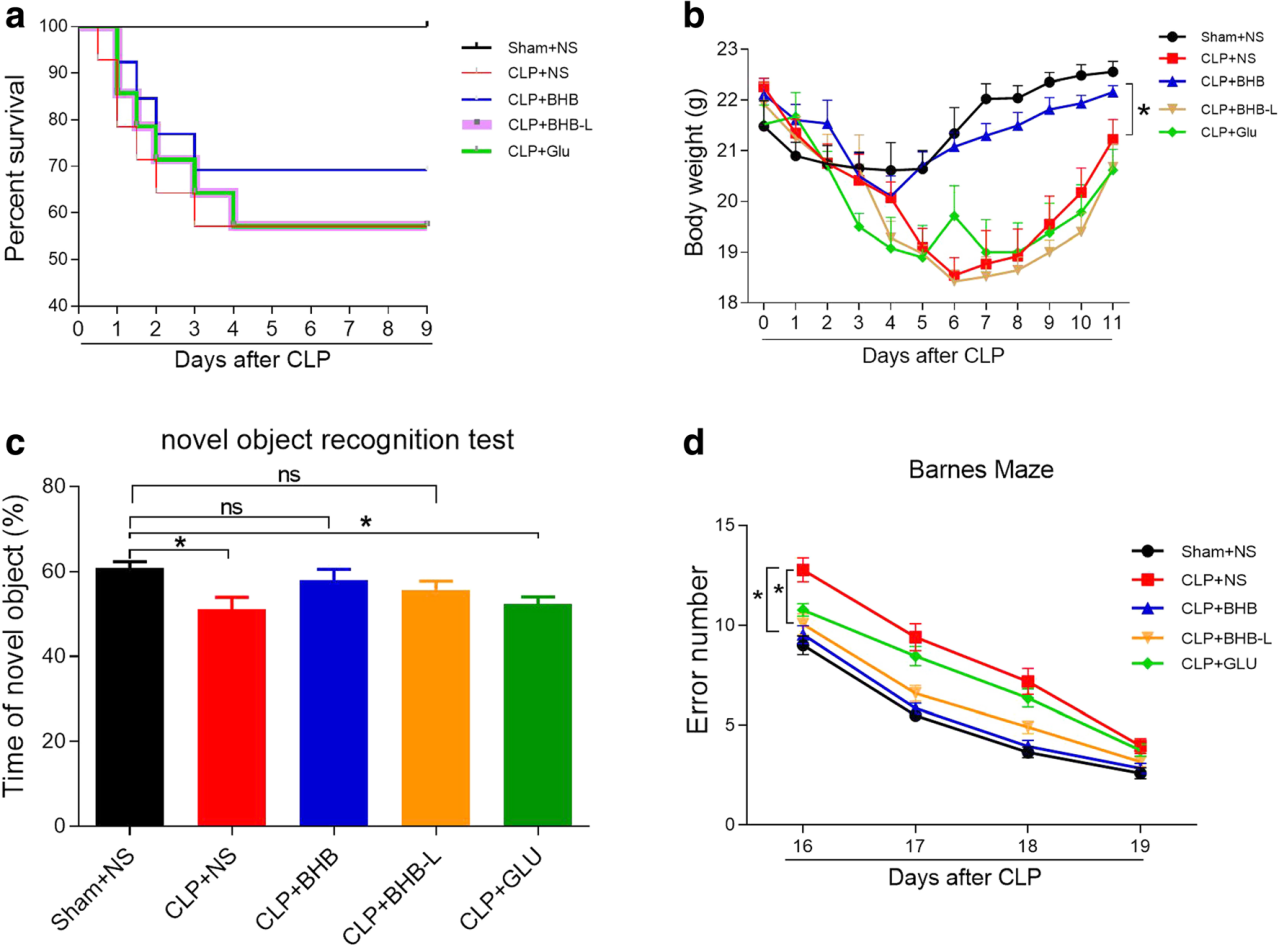
β-hydroxybutyrate(BHB) administration promotes survival and body weight recovery of CLP mice and improves cognitive dysfunction of CLP surviving mice (A) The survival of each group during the first 9 days after CLP surgery. (B) The body weight recovery of each group during the first 11 days after CLP surgery. *p =0.0003 vs CLP+NS group. (C) In the novel object recognition test 14−15 days after CLP, the time of the novel object of the CLP+NS and CLP+Glu groups were significantly less than that of the sham+NS group, but not of the CLP+BHB and CLP+BHB(L) groups (p>0.05). *p<0.05 vs sham + NS group. ns: p>0.05 vs sham + NS group. (D) In the Barnes maze test 16−19 days after CLP, compared to the CLP+NS group, the error number in CLP+BHB (p=0.002) and CLP+BHB(L) (p=0.045) groups decreased significantly, but the CLP+Glu (p=0.657) group did not. *p<0.05 vs LPS+NS group. N = 9-13/group. The data are expressed as the mean±SEM. sham+NS= sham + saline injection group. CLP+NS= CLP surgery+ saline injection group. CLP+BHB = CLP surgery+ BHB injection group. CLP+BHB (L)= CLP surgery + BHB injection of late stage group. CLP+Glu = CLP surgery+ glucose solution injection group. CLP= cecal ligation and perforation surgery

To investigate whether BHB could prevent post-sepsis cognitive impairment and affect the severity of sepsis directly, we detected the effects of BHB on survival, body weight recovery, learning, and memory of CLP mice. Compared to the CLP+NS group, the CLP+BHB group had faster recovery of body weight (p = 0.0003) (Fig. 1B), and a tendency of higher survival rate (Fig. 1A). In the novel object recognition test, the time of the novel object of the CLP+NS and CLP+Glu groups was significantly less than that of the sham+ NS group (Sham+NS vs. CLP+NS, p = 0.024; Sham+NS vs. CLP+GLU, p = 0.035), but CLP +BHB and CLP +BHB(L) groups did not (p>0.05, respectively) (Fig. 1C). In the Barnes maze test, compared to the CLP+NS group, the error number in CLP+BHB (p = 0.002) and CLP+BHB(L) (p = 0.045) groups decreased significantly, but the CLP+Glu (p = 0.657) group did not (Fig. 1D). These data showed that BHB administration increased the survival and the body weight recovery of sepsis mice and improved the learning and memory of sepsis surviving mice. The glucose solution treatment did not show similar effects on the septic mice.

### Subcutaneous administration of BHB increased the BHB level of blood and brain of CLP mice

**Fig. 2:**
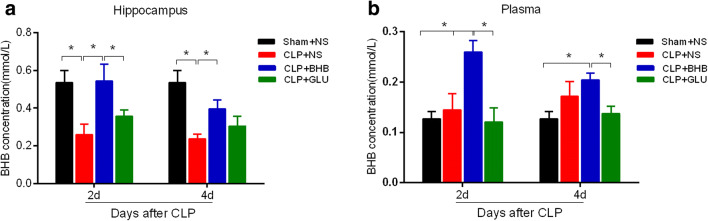
Subcutaneous administration of BHB increased the BHB level of blood and brain of CLP mice (A) The BHB concentration in the hippocampus of CLP mice on the 2^nd^ and 4^th^ days after CLP surgery. *p< 0.05. (B) The BHB concentration of blood plasma in CLP mice on the 2^nd^ and 4^th^ days after CLP surgery. *p< 0.05. N = 3-5/time point/group. The data are expressed as the mean±SEM. sham+NS= sham + saline injection group. CLP+NS= CLP surgery+ saline injection group. CLP+BHB = CLP surgery+ BHB injection group. CLP+BHB-L= CLP surgery + BHB injection of late stage group. CLP+Glu = CLP surgery+ glucose solution injection group. CLP= cecal ligation and perforation surgery

In order to identify whether subcutaneously applied BHB entered blood and brain, we detected the level of BHB in the blood and hippocampus of CLP mice. Compared to the sham+NS group, the BHB level of the hippocampus in the CLP+NS group significantly decreased on the 2^nd^ and 4^th^ days after CLP (p<0.05, respectively), which was obviously alleviated in the CLP+BHB group (p<0.05, respectively) (Fig. 2). The level of blood BHB in the CLP+BHB group was significantly higher than the CLP+NS group on the 2^nd^ day after CLP (p<0.05, respectively) (Fig. 2). These results showed that subcutaneously applied BHB could enter blood and brain.

### BHB administration improved neuroplasticity in CLP surviving mice

**Fig. 3:**
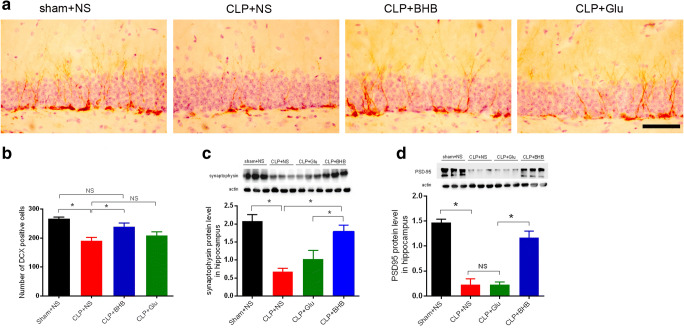
BHB administration improved neuroplasticity in CLP surviving mice (A) The representative images and quantitative analysis of doublecortin positive cells in the dentate gyrus of CLP mice 12 days after CLP surgery. *p< 0.05. N = 4/group. Bar=50 μm. (B) and (C) Representative Western blot analysis of synaptophysin and PSD-95 in the hippocampus 12 days after CLP surgery. * P< 0.05. N = 3/group. The data are expressed as the mean±SEM. sham+NS= sham + saline injection group. CLP+NS=CLP surgery+ saline injection group. CLP+BHB = CLP surgery+ BHB injection group. CLP+Glu = CLP surgery+ glucose solution injection group. CLP= cecal ligation and perforation surgery

Neuroplasticity is the structural basis of learning and memory. Thus, we detected whether, corresponding to the improvement of learning and memory, BHB administration also induced changes of hippocampus neuroplasticity of CLP surviving mice just before starting the behavior test. Neuroplasticity was marked by synaptic protein synaptophysin and PSD-95 and the adult neurogenesis marker doublecortin (DCX). Compared to the sham+NS group, the expressions of synaptophysin and PSD-95 and the number of DCX+ cells all decreased in the hippocampus of the CLP+NS group on the 12^th^ day after CLP (p<0.05, respectively), which was significantly improved in the CLP+BHB group (p<0.05, respectively), but not in the CLP+Glu group (p>0.05, respectively) (Fig. 3). These results showed the improvement of BHB to neuroplasticity of CLP surviving mice.

### BHB administration limited neuroinflammation and peripheral inflammation in CLP mice

**Fig. 4:**
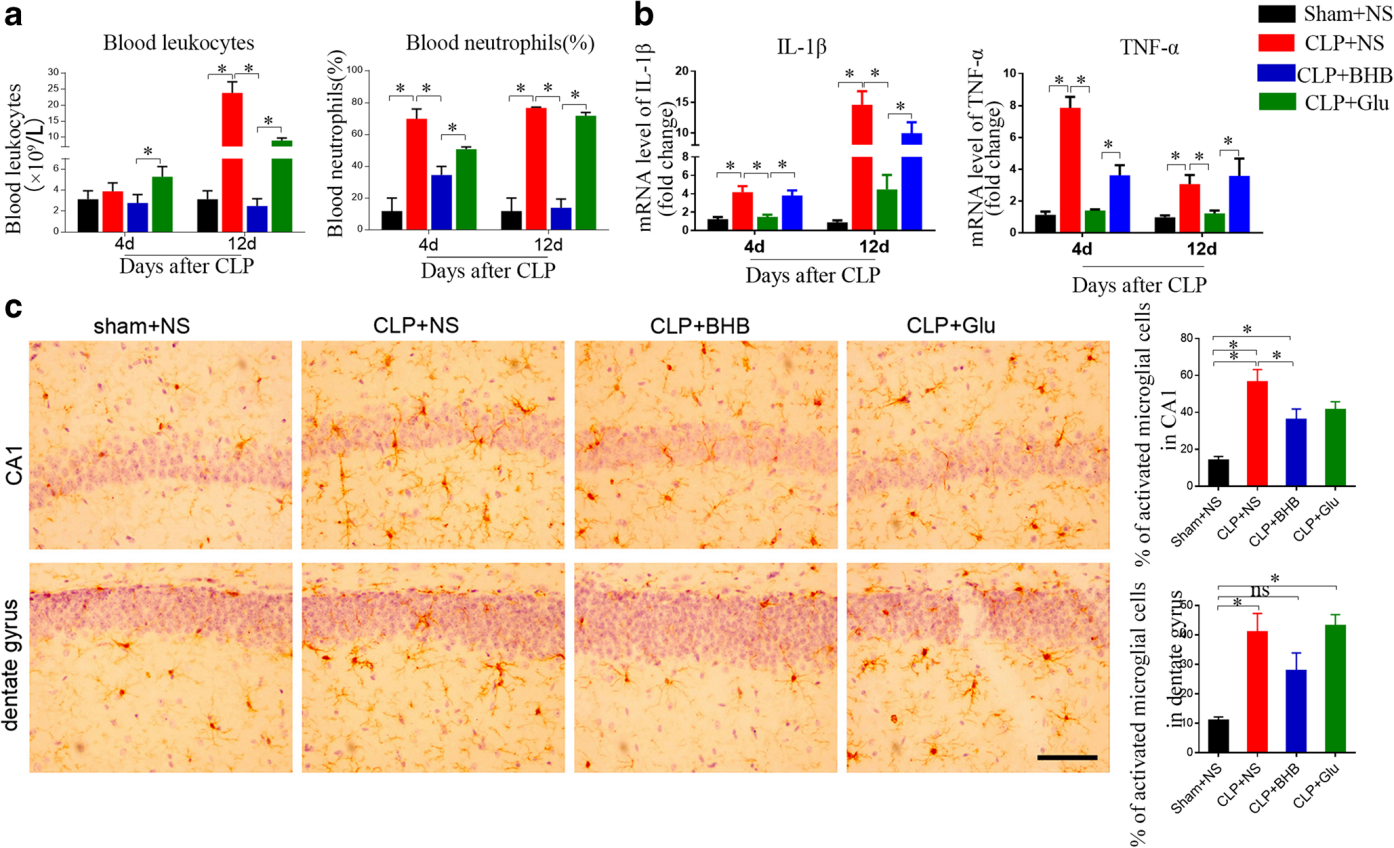
BHB administration limited neuroinflammationin and peripheral inflammation in CLP mice (A) Sepsis-induced changes of the leukocyte number and neutrophil percentage of peripheral blood were significantly limited by BHB. *p< 0.05. N = 4-5/time point/group. The data are expressed as the mean±SEM. (B) Levels of the inflammatory factors IL-1β and TNF-α in the hippocampus. *p< 0.05. N = 4-5/time point/group. (C) The representative images of Iba1 staining and a quantitative analysis of activated microglial cells in CA1 and the dentate gyrus of CLP mice 12 days after CLP surgery. *p < 0.05. Bar = 50 μm. sham+NS = sham + saline injection group. CLP+NS= CLP surgery+ saline injection group. CLP+BHB = CLP surgery+ BHB injection group. CLP+Glu = CLP surgery+ glucose solution injection group. CLP = cecal ligation and perforation surgery

Neuroinflammation plays an important role in pathogenesis of post-sepsis cognitive impairment [1, 8–13]. To test the possible mechanism that BHB prevented post-sepsis cognitive impairment, we detected the effects of BHB on neuroinflammation in CLP. Compared to the sham+NS group, the mRNA levels of hippocampal IL-1β and TNF-α in the CLP+NS group significantly increased on the 4^th^ and 12^th^ days after CLP (p<0.05, respectively) (Fig. 4B). And the mRNA levels of hippocampal IL-1β and TNF-α in the CLP+BHB group were significantly lower than the CLP+NS and the CLP+Glu groups (p<0.05, respectively) (Fig. 4B). In addition, the microglia activation percentages in CA1 and dentate gyrus of the CLP+NS group were obviously higher than those of the sham +NS group on the 12^th^ day after CLP (p<0.05, respectively), which was significantly limited in the CLP+BHB group (Fig. 4C). These results showed that BHB administration reduced neuroinflammation in CLP mice, but a glucose solution treatment had little effect.

We also detected the effects of BHB on the peripheral inflammation in CLP mice. Compared to the sham+NS group, the percentage of blood neutrophils in CLP+NS group significantly increased on the 4^th^ and 12^th^ days after CLP (p<0.05, respectively), and the amount of blood leukocytes in the CLP +NS group also significantly increased on the 12^th^ day, which was obviously reduced in the CLP+BHB group (Fig. 4A). In contrast, the percentage of blood neutrophils and the amount of blood leukocytes in the CLP+Glu group were higher than the CLP+NS group (p>0.05, respectively) (Fig. 4A). These results showed that BHB administration reduced neuroinflammation and peripheral inflammation in CLP mice, but a glucose solution treatment had little effect.

### Intracerebroventricular administration of BHB improves cognitive dysfunction and reduces neuroinflammation in CLP mice

**Fig. 5:**
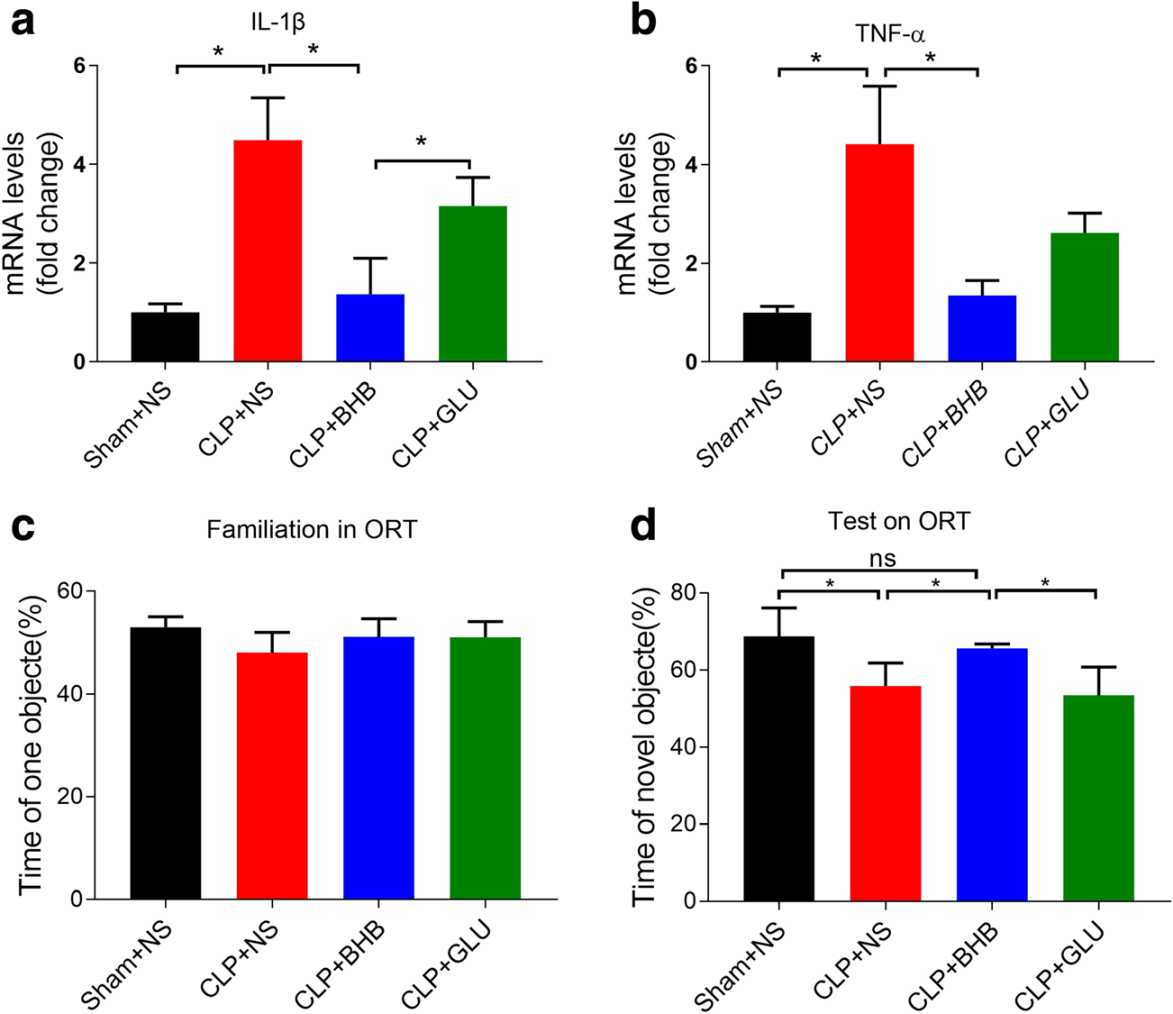
Intracerebroventricular administration of BHB improves cognitive dysfunction and limits neuroinflammation in CLP mice (A) and (B) Levels of the inflammatory factors IL-1β and TNF-α in the hippocampus 12 days after CLP . *p< 0.05. N = 4/group. The data are expressed as the mean±SEM. (C) and (D) In the novel object recognition test 11-12 days after CLP, the time of the novel object of the CLP+NS and CLP+Glu groups were significantly less than the CLP +BHB group during the test phase, although there was no intergroup difference during the familiarization phase. *p<0.05. N = 10−14 / group. The data are expressed as the mean±SEM. ns=no significance. sham+NS = sham + saline injection group. CLP+NS= CLP surgery+ saline injection group. CLP+BHB = CLP surgery+ BHB injection group. CLP+Glu = CLP surgery+ glucose solution injection group. CLP = cecal ligation and perforation surgery

In order to test where BHB functioned in protecting the brain of CLP mice, we evaluated the effects of intracerebroventricular administration of BHB on the cognitive function and the neuroinflammation of CLP mice. Compared to the CLP+NS and CLP+Glu groups, the mRNA levels of hippocampal IL-1β and TNF-α decreased significantly and the exploration time devoted to the novel object in the novel object recognition test increased significantly in the CLP+BHB group (p < 0.05, respectively) (Fig. 5). These data showed that BHB could protect the brain of CLP mice in a direct intrabrain manner.

### BHB administration limited LPS-induced neuron damage and inflammatory response via HCA2 and MCT2 in vitro

**Fig. 6:**
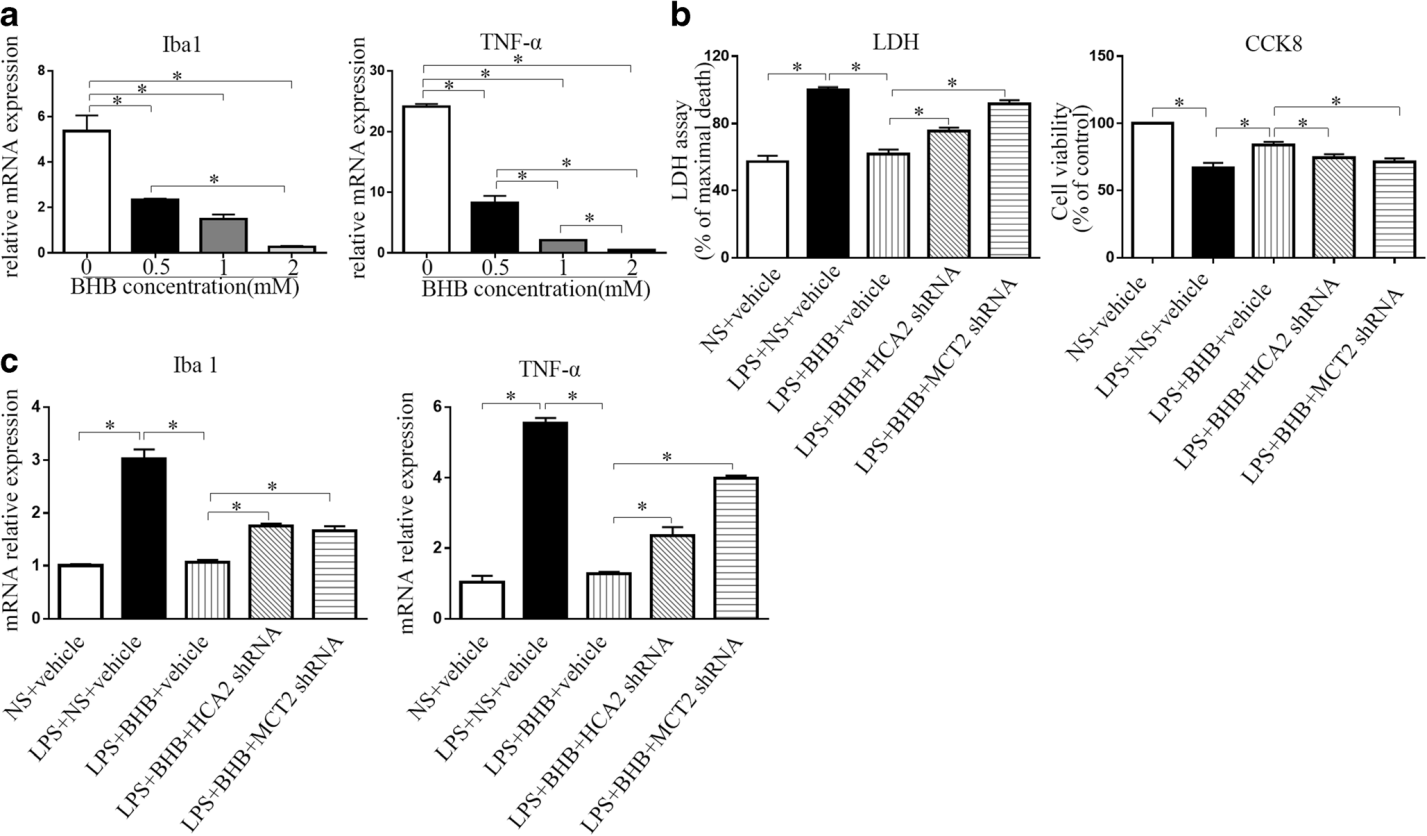
BHB administration limited LPS-induced neuron damage and inflammatory response via HCA2 and MCT2 in the coculture of neurons, astrocytes, and BV2 (A) A quantitative RT-PCR analysis showed that BHB inhibited the LPS-induced changes of the inflammatory markers Iba1 and TNF-α in microglial cells in a dose-dependent manner. *p< 0.05. N = 3/group. (B) Neuronal damage was marked by the levels of medium LDH and neuronal CCK-8. LPS induced obvious neuron damage, which was significantly limited by BHB. *p< 0.05. N =3-4/group. (C) BHB alleviated LPS-induced inflammatory response. Additionally, knocking down the levels of HCA2 of microglial cells and MCT2 of neurons both obviously blocked the protective effects of BHB on the inflammatory response. *p< 0.05. N =3-4/group. The data are expressed as the mean±SEM. NS= normal saline. LPS= lipopolysaccharides. HCA2 shRNA = lentivirus (LV) containing shRNA plasmid targeting HCA2. MCT2 shRNA= lentivirus (LV) containing shRNA plasmid targeting MCT2

Previous studies have showed that HCA2 is a BHB specific receptor and is expressed mainly in microglial cells [22]. MCT2 is a BHB transporter and is expressed mainly in neurons [23]. To test the possible molecular mechanism by which BHB limited neuroinflammation and improved SAE, we first detected the dose effects of BHB (0.5, 1.0, and 2.0 mM) on the LPS (1 μg/ml)-induced inflammatory response in the coculture of neurons, astrocytes, and BV2 cells (a microglial cell line) (Fig. 6A). We found that BHB could significantly decrease the mRNA levels of Iba1 and TNF-α in a dose-dependent manner (p<0.05, respectively) and that 1.0 mM BHB was considerably more suitable for a mechanism study (Fig. 6A). In order to find specific shRNAs for neuronal MCT2 and microglial HCA2, we evaluated the efficiencies of six shRNAs by transfecting BV2 cells (for HCA2 ) or primary neurons (for MCT2 ) with a lentivirus, and picked out the effective shRNAs for MCT2 and HCA2 (Fig. S1). Based on these, we detected the effects of MCT2 and HCA2 knockdown on the protection of BHB administration in a LPS (1 μg/ml)-treated coculture of neurons, astrocytes, and BV2 cells. Compared to the control, the LPS treatment significantly increased the level of LDH of the culture medium, decreased neuron viability (marked by CCK8), and induced a strong inflammation response (p<0.05, respectively) (Fig. 6B and C). The BHB administration obviously alleviated the effects of LPS on the LDH level, neuron viability, and inflammation response (p<0.05, respectively) (Fig. 6B and C). Knocking down MCT2 of neurons and HCA2 of BV2 cells by specific shRNA both partly decreased the protection of BHB to neurons (Fig. 5B and C) and the inhibition of BHB to inflammation response (Fig. 6C). Additionally, knocking down HCA2 of BV2 cells by specific shRNA counteracted the effects of BHB on inflammation response more obviously than did a MCT2 knockdown of neurons (Fig. 6B and C). These results showed that HCA2 and MCT2 both played roles in the improvement of BHB to LPS-induced neuron damage and inflammatory response, and HCA2 played a more important role in inflammatory response than MCT2.

## Discussion

The prevention of post-sepsis cognitive impairment remains clinically challenging. Aside from anti-infection treatments and goal-directed supportive treatments at the early stage of sepsis, no specific method has been generally accepted for its prevention. In this study, we aimed to investigate the effects and underlying mechanisms of exogenous β-hydroxybutyrate (BHB) on post-sepsis cognitive impairment with the ultimate goal of developing a pharmacological adjunct treatment for post-sepsis cognitive impairment. We found that BHB administration increased the survival and the body weight recovery of sepsis mice and improved the learning and memory of sepsis surviving mice in a cecal ligation and perforation-induced sepsis model (Fig. 1). Corresponding to the cognitive improvement, BHB administration also alleviated sepsis-induced neuroplasiticity damage (Fig. 3). In contrast, the glucose solution treatment did not show similar effects on the sepsis mice. These data are consistent with previous reports [7, 24]. Julio-Amilpas et al. reported that BHB administration could prevent hypoglycemia-induced neuronal death in rats [7]. And Steckert et al. reported that a single intraventricular injection of sodium butyrate before cecal ligation and perforation surgery could improve the impairment of aversive memory of sepsis surviving rats [24]. More interestingly, in the study, we also detected significant protective effects on post-sepsis cognitive impairment even if BHB was administrated at the late stage of sepsis (Fig. 1). These above data showed that no matter when BHB was administrated during sepsis, it could alleviate post-sepsis cognitive impairment without obvious side effects.

Ketone body β-hydroxybutyrate (BHB) is an alternative energy source for the brain during a state of energy deficit. In order to investigate the mechanisms underlying the prevention of BHB on the post-sepsis cognitive impairment, we first detected the ratio of ADP/ATP of hippocampus during sepsis. We found that BHB administration didn’t induce a significant change of ADP/ATP ratio of hippocampus in CLP mice (Fig. S2), suggesting that BHB administration prevented post-sepsis cognitive impairment via not providing energy. Neuroinflammation is the most accepted pathological mechanism of post-sepsis cognitive impairment. Thus we evaluated the effects of BHB on neuroinflammation. We found that (1) peripheral administration of BHB reduced neuroinflammation and peripheral inflammation in CLP mice, (2) intracerebroventricular administration of BHB also limited neuroinflammation in CLP mice, and (3) peripheral administration of BHB increased the BHB level of blood and brain of CLP mice. These showed that peripheral administration of BHB could limit neuroinflammation directly and indirectly through a decrease in systemic inflammation. Moreover, in the coculture of neurons, astrocytes, and BV2 cells (a microglial cell line), BHB treatment alleviated lipopolysaccharides-induced inflammatory response and neuron damage (Fig. 6). And HCA2 of BV2 cells played more important roles in anti-inflammation of BHB than MCT2 of neurons (Fig. 6). This information showed that anti-neuroinflammation was an important mechanism underlying the prevention of BHB to post-sepsis cognitive impairment. Previous studies showed that BHB was an endogenous histone deacetylase inhibitor [18], and sepsis induced obvious changes of histone deacetylases [25–27]. Thus, histone deacetylases were the possible downstream molecules of HCA2 and MCT2. And the exact molecule signals need further investigation.

Taking together, our data suggested that BHB was a potential pharmacological adjunct treatment for prevention of post-sepsis cognitive impairment. And inhibiting neuroinflammation via HCA2 was an important mechanism.

## Electronic supplementary material

Fig. S1 shRNA interference of MCT2 and HCA2 (A) and (B) Bright field and GFP-positive neurons infected with lenti-virus shMCT2. (C) The statistics of three shRNA strand intervention levels of shMCT2 (target sequence of sequence 1−3: TTCATTGGAGGTTTAGGATTA, CTGAAGAAAGCCAGTAAGGTA, or CAGGACGAAGTTACTGTCAAA); we chose sequence 1 for further studies. (D) and (E) Bright field and GFP-positive neurons infected with lenti-virus shHCA2. (C) The statistics of three shRNA strand intervention levels of shHCA2 (target sequence of sequence 1−3: CAACAAATACCAGATGGTT, CCAACATTTCGTAGCCTTA, or TCAGATGAACGACGTTATT); we chose sequence 3 for further studies. The data are presented as the mean±SEM. *p<0.05 (JPG 4158 kb)

Fig. S2 BHB administration did not obviously change the energy state of the hippocampus in CLP mice 4 days after CLP NS: no significance. N=4/group. sham+NS = sham + saline injection group. CLP+NS= CLP surgery+ saline injection group. CLP+BHB = CLP surgery+BHB injection group. CLP+Glu = CLP surgery+ glucose solution injection group. CLP = cecal ligation and perforation surgery (JPG 28 kb)

ESM 3 (PDF 1224 kb)
