# Supplementary figures and images for: Subcutaneous administration of β-hydroxybutyrate improves learning and memory of sepsis surviving mice
Source: Neurotherapeutics. 2019 Dec 18;17(2):616–26. doi: 10.1007/s13311-019-00806-4 (PMC7283433; doi:10.1007/s13311-019-00806-4)

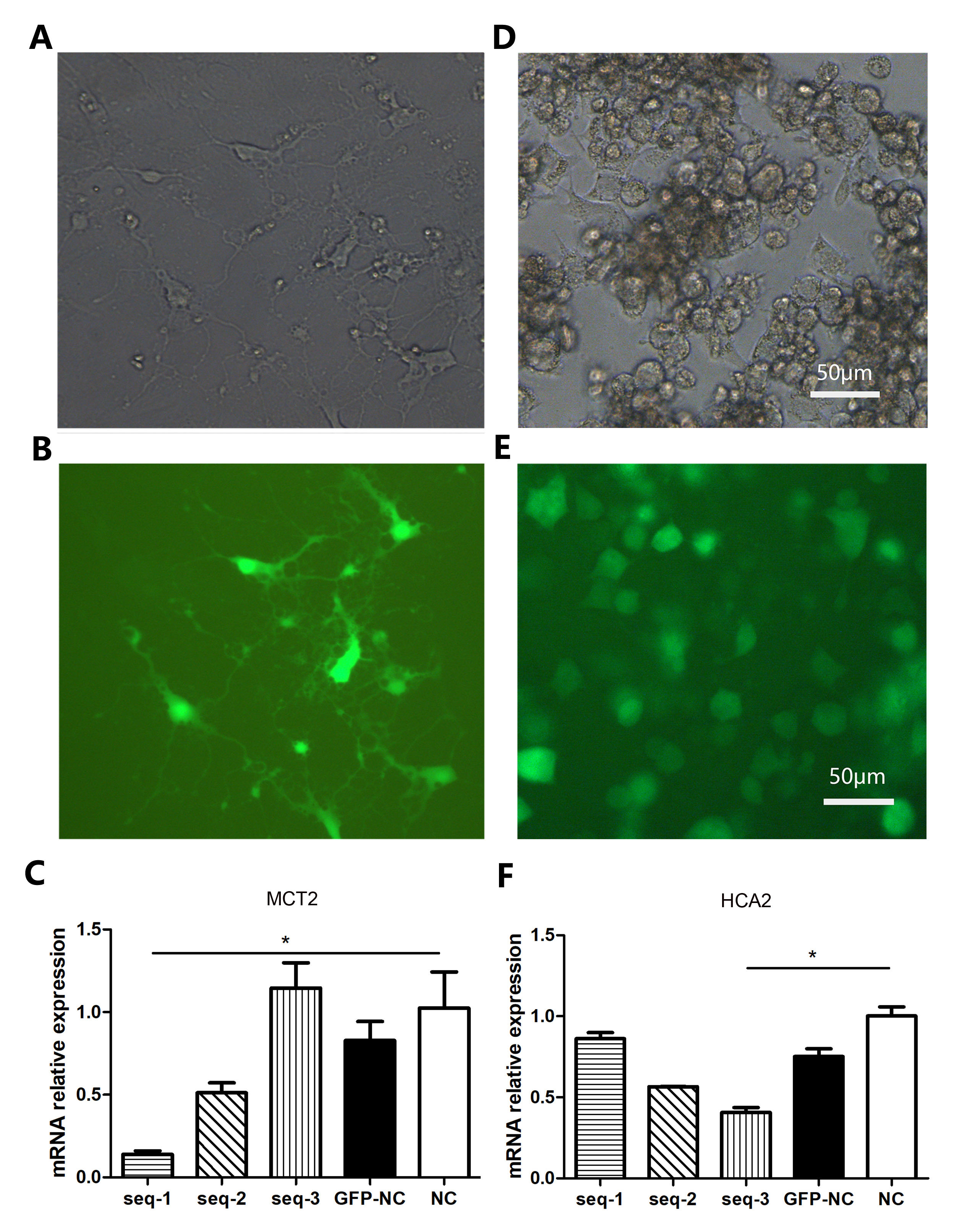

Supplement: Supplementary file 1 — shRNA interference of MCT2 and HCA2 (A) and (B) Bright field and GFP-positive neurons infected with lenti-virus shMCT2. (C) The statistics of three shRNA strand intervention levels of shMCT2 (target sequence of sequence 1−3: TTCATTGGAGGTTTAGGATTA, CTGAAGAAAGCCAGTAAGGTA, or CAGGACGAAGTTACTGTCAAA); we chose sequence 1 for further studies. (D) and (E) Bright field and GFP-positive neurons infected with lenti-virus shHCA2. (C) The statistics of three shRNA strand intervention levels of shHCA2 (target sequence of sequence 1−3: CAACAAATACCAGATGGTT, CCAACATTTCGTAGCCTTA, or TCAGATGAACGACGTTATT); we chose sequence 3 for further studies. The data are presented as the mean±SEM. *p<0.05 (JPG 4158 kb) [file 13311_2019_806_MOESM1_ESM.jpg]

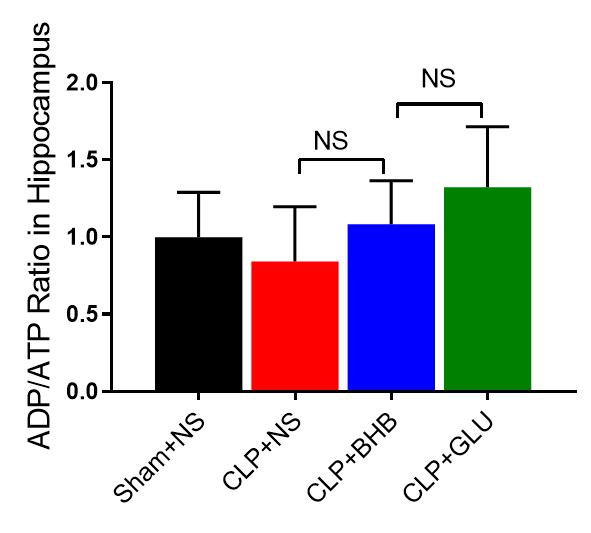

Supplement: Supplementary file 2 — BHB administration did not obviously change the energy state of the hippocampus in CLP mice 4 days after CLP NS: no significance. N=4/group. sham+NS = sham + saline injection group. CLP+NS= CLP surgery+ saline injection group. CLP+BHB = CLP surgery+BHB injection group. CLP+Glu = CLP surgery+ glucose solution injection group. CLP = cecal ligation and perforation surgery (JPG 28 kb) [file 13311_2019_806_MOESM2_ESM.jpg]
